# A Newly Developed Orthodontic-Logopedic Screening Procedure for Myofunctional Dysfunctions—A Pilot Study

**DOI:** 10.3390/jpm13081248

**Published:** 2023-08-10

**Authors:** Christoph-Ludwig Hennig, Antonia Neumann, Ann Nitzsche, Albert Stemmler, Knuth Tränckner, Nicola Kühn, Thomas Lehmann, Collin Jacobs

**Affiliations:** 1 Department of Orthodontics, Center of Dental Medicine, Jena University Hospital, An der Alten Post 4, 07743 Jena, Germany; 2 Praxis of Orthodontics Dr. Knuth Tränckner, Schenckendorfstraße 1, 07318 Saalfeld, Germany; 3 Praxis of Orthodontics Nicola Kühn, Alexander-Puschkin-Platz 3, 99510 Apolda, Germany; 4 Institute of Medical Statistics, Computer Science and Data Sciences, University of Jena, Bachstraße 18, 07743 Jena, Germany

**Keywords:** interdisciplinary orthodontics, patient-personalized orthodontic treatment, new orthodontic-logopedic screening procedure, myofunctional dysfunction

## Abstract

Interdisciplinary, patient-specific cooperation between orthodontics and speech therapy plays an important role in the therapy of myofunctional dysfunctions. The following orthodontic-logopedic screening procedure is intended to objectify the diagnosis of such dysfunctions and the progress of therapy. A diagnostic questionnaire was prepared based on existing diagnostic questionnaires for myofunctional dysfunction. It contains 32 questions, with a clinical weighting of 0 to 50 points in total. This results in a point score. The lower the score is, the lower the need for therapy is. The study included 108 patients between the ages of 6 and 50 years. After screening, the patient population was divided into Group 0 (score < 15; no speech therapy need; *n* = 36) and Group 1 (score ≥ 15; a speech therapy need; *n* = 72). Group 1 was additionally randomized into Subgroups A (with speech therapy; *n* = 36) and B (without speech therapy; *n* = 36). After a treatment interval of 6 months, all patients in Group 1 were examined again with the help of the screening procedure. Statistical analysis (SPSS) and significance testing (Mann–Whitney U test) were performed. At baseline, there was no significant difference between patients in Subgroups A and B (*p* = 0.157). Subgroup A had a median score of 25, and Subgroup B had a median score of 30. However, after the treatment interval, a significant improvement (*p* = 0.001) for Subgroup A with a median score of 11 (mean score difference = 14.67) over Subgroup B with a median score 23 (mean score difference of 7.08) was observed. The developed screening procedure was shown to be equally applicable to all patients and treatment providers. With the help of the scores in point form, the need for speech therapy and the progress of such therapy can be objectified.

## 1. Introduction

Myofunctional dysfunction (MD), or orofacial dysfunction, refers to the faulty swallowing of a child, adolescent, or adult [1]. The tongue does not assume its physiological position during the swallowing process but, rather, is pushed forward or laterally between or against the teeth. This continuous tongue-pressing or tongue-thrust has an enormous force effect on the teeth when several thousand swallows are made per day [1]. Furthermore, MD is described as a “disturbance of muscle function, muscle tone, and physiological movement patterns in the orofacial region” [2]. The orofacial musculature becomes flaccid. The tone of the overall body musculature also decreases, as does diaphragmatic tension [1]. It is observed in addition to a lack of muscular force, as well as the absence of physiological pressures in the oral-nasopharynx, which have a formative influence on the jaw and dentition. Dysgnathia is often the result [2]. Of course, an unfavorable tooth or jaw position also has a negative effect on myofunction. Thus, the progenic dentition usually leads to a pathological resting position for the tongue on the floor of the mouth, from which swallowing also takes place. The tongue presses against the lower incisors during the swallowing process, which further promotes Class III dentition. In addition to the open bite mentioned above, the cross bite also has a negative effect on orofacial balance [1]. However, whether dysgnathia affects the facio-oral musculature more strongly or MD, conversely, affects tooth and jaw position more strongly can likely never be conclusively determined. Therefore, it should be considered as an inevitable interaction of the craniofacio-oral system. Mild MD can also be caused by orthodontic appliances [3]. For example, the smooth acrylic of maxillary plates tempts the tongue to slide forward. In addition, the activator, for example, offers little space for the tongue, which means that palatal contact cannot be established optimally [3].

Anatomical anomalies, such as a shortened or attached lingual frenulum, true macroglossia (e.g., in Down’s disease), tonsillar hyperplasia, or cleft lip and palate, naturally affect orofacial function and, thus, tongue mobility, tongue tone, lip closure, and intraoral pressures. It is difficult to diagnose MD objectively and assess and measure the success of therapy. Currently, there are various techniques used to diagnose MD in orthodontics and speech therapy, but there is no interdisciplinary concept in this regard. This study presents an objective orthodontic-logopedic screening procedure that addresses individual patient symptoms and therapy. A newly developed orthodontic-logopedic screening questionnaire will be used to help improve the diagnosis and holistic care of patients with orofacial dysfunction. For this purpose, a systematic search in online databases and a manual search in reference books was performed to review and subsequently optimize the available diagnostic and screening material. Because, in general, patients with dysgnathia often have orofacial dysfunctions, it is important to provide them with supportive myofunctional therapy. To ensure this in borderline cases or cases of hidden orofacial problems but also not overlook any patients and, thus, potentially risk a recurrence, a standardized screening procedure should be used. It should cover the overlapping fields of orthodontics and speech therapy and be applicable to all patients in orthodontic and speech therapy practice. It is important that the screening sheet can be applied equally by both departments and that a usable result can be achieved. The aim of this study was to develop an interdisciplinary and individualized screening questionnaire for myofunctional dysfunction, which serves to objectify the diagnosis and the course of therapy.

## 2. Materials and Methods

### 2.1. Development of the Screening Sheet

The “new orthodontic-logopedic screening procedure for myofunctional dysfunctions” was developed based on pre-existing findings and diagnostic questionnaires for myofunctional disorders. Because only one of the existing diagnostic questionnaires met the requirements to be used in speech therapy, as well as in other specialties, such as orthodontics [4], we decided to create a new screening questionnaire. First, a search of published diagnostic questionnaires was conducted. For this purpose, a systematic search in the database of the Thuringian University and State Library (ThULB), in the American database Pubmed (NLM Pubmed), the online portal for logopedic therapy material www.madoo.net, and a manual search in reference books and therapy material for logopedic practice was performed. The search covered the period from mid-May 2021 to mid-October 2021. The terms “myofunctional disorder”, “orofacial dysfunction”, “orthodontics”, and “diagnostics” were used in the ThULB database, and the terms “orthodontics”, “orofacial dysfunction”, “deglutition disorders”, “speech therapy”, and “diagnostics” were used in Pubmed. The terms were linked using the operators “OR” and “AND”. Large numbers of studies and articles were found in the ThULB database, as well as in Pubmed. However, few of them contained a research questionnaire. Table 1 summarizes the studies found via the search that had a report card (Table 1). It also lists the name and author of the investigation forms that were used to develop the new orthodontic logopedic screening procedure. Particular emphasis was placed on ensuring that the screening is understandable and feasible for practitioners in both departments. With the help of a point system, it should be possible to achieve an unambiguous result. It was also important to us that, in addition to the classic examination methods for swallowing dysfunction, such as swallowing saliva and water with open lips, the visualization of swallowing using the Payne technique and the orthodontic parameters are also included in the diagnostics. The newly developed orthodontic-logopedic screening sheet would be developed jointly by a speech therapist and an orthodontist. Components of already existing screening sheets were optimized, individualized and made feasible for interdisciplinary use. Likewise, care was taken to ensure that this could be implemented in everyday work and tested in the meantime.

### 2.2. Structure of the Screening Sheet

**Figure 1:**
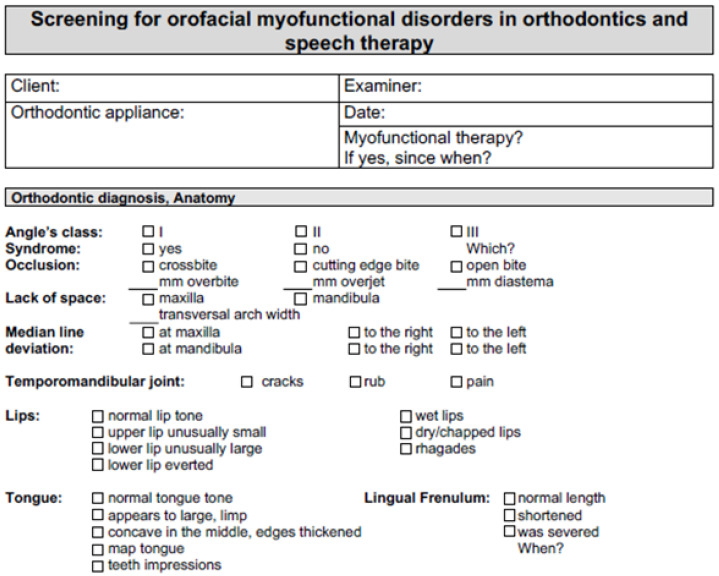
The part of the interdisciplinary orthodontic-logopedic screening sheet with the patient data mask and the orthodontic diagnosis and anatomy section.

In addition to the patient data mask, the screening sheet is divided into sections for orthodontic diagnosis, anatomy, and the indications of a myofunctional swallowing disorder before logopedic treatment and, as a progress control, after logopedics. Thus, during an initial examination, only the first two pages are completed; the remainder are left until the follow-up control. In the best case, this takes place after the logopedic treatment has been completed or after about half a year. The patient data mask contains the patient’s master data (name and date of birth), the name of the examiner, the date of the initial examination, the current orthodontic treatment of the patient to be examined, and the information concerning whether the patient has already had logopedic therapy. The orthodontic diagnosis and anatomy section contains the most important orthodontic parameters, such as the angle class and the occlusion, including information on overjet, overbite, and diastema mediale in millimeters. This section records whether there is a space deficiency, an abnormality of the transversal dental arch width, or a midline shift (median line deviation) in one or both jaws. In addition, information can be given regarding previous syndromal disease, known temporomandibular joint disorders, and other special features, such as the non-attachment of teeth or similar features. Furthermore, a statement can be recorded about the anatomy of the lips, tongue, and lingual frenulum and overall body tonus (Figure 1).

For the description of the lip and tongue anatomy, the physiological appearance and appearances typical of myofunctional disorders are given as selections. These can be recorded by simply marking them with a cross. In addition to myofunctional disorders, the phonation of the tongue fricatives is also diagnosed, and swallowing is visualized using the Payne technique. A spatula is used to apply fluorescent Payne paste to various areas of the tongue.

The tongue is brought in, then a mirror is used to check whether impressions of the tongue position are visible on the palate at rest. Then, the patient swallows. The imprints of the Payne paste on the palate and tongue indicate how the tongue moved at the moment of swallowing and whether there is an abnormal swallow. The Payne lamp can be used to clearly visualize the imprints of the fluorescent Payne paste by shining a light through a mirror onto the palate. The swallow marks can be seen. In the resting position section, the lips and the facial/chewing muscles can be recorded in their resting position. The muscles are described as active or inactive. The lips can be physiologically loosely closed (i.e., “competent” or “potentially competent” (opened from time to time or tightly compressed and loosely closed), “tightly closed” (often showing activity on the part of the mentalis muscle), or “incompetent” (lips are open) or the upper anterior teeth may bite on the lower lip (Figure 1).

**Figure 2:**
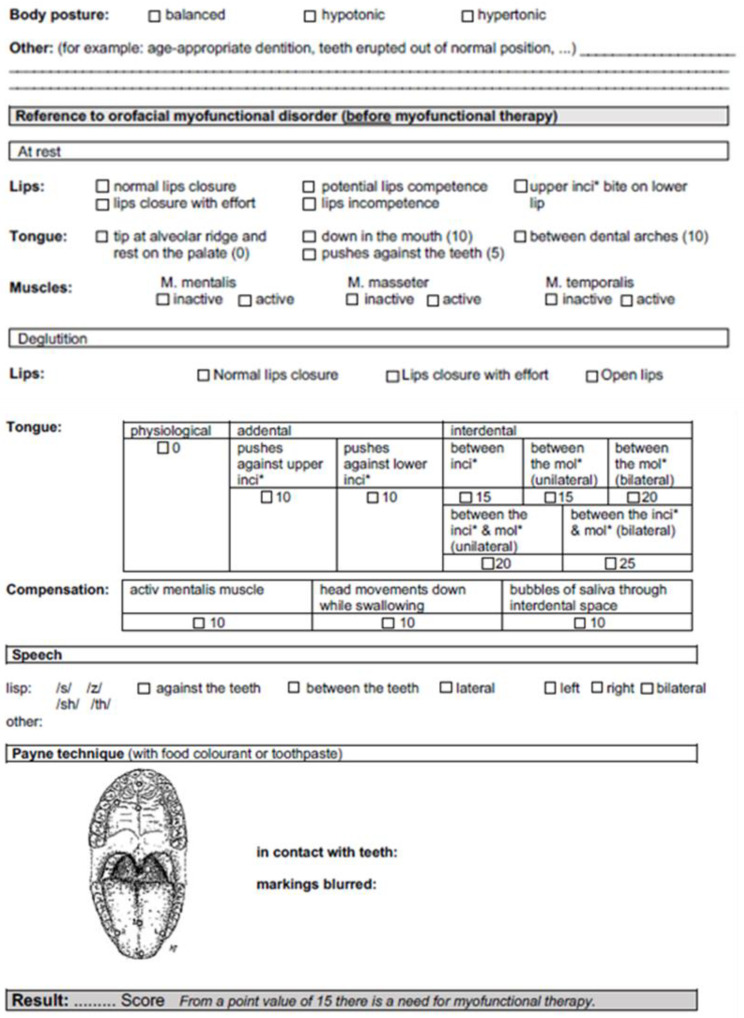
The part of the interdisciplinary orthodontic-logopedic screening sheet with references to orofacial myofunctional disorders, the tongue position, tongue function, the speech function, and the swallowing section.

The position of the tongue can be described as “from the alveolar margin on the palate” (corresponding to the physiological position), “down of the mouth”, “addental”, or “interdental.” A point value is assigned for each of these tongue positions. This is based on the severity of the pathology involved. For example, if the tongue’s resting position is described as being both on the floor of the mouth and pressed against/between the upper or lower anterior or posterior teeth, the respective point values can be added together (Figure 2).

The swallowing section refers to lip and tongue position and compensation mechanisms during swallowing. With respect to the lips, the typical positions can be physiologically recorded as “loosely closed”, “pressed tightly together”, or “open.” During swallowing, the tongue can assume various pathological positions, in addition to the physiological position. These are also noted on the sheet and assigned a point value. If several pathologies occur, several crosses can be made, and the corresponding point values can be added together. In addition to a faulty tongue position, compensation mechanisms are frequently observed in patients with myofunctional swallowing disorders. These serve to support the closure of the mouth (“mentalis muscle active”), support the undulation of the tongue to trigger the swallowing reflex (“downward head movement during swallowing”), or testify to the insufficient sealing of the tongue during the swallowing process (“pushes saliva vesicles through interdental spaces”). These compensations were also assigned a point value. Furthermore, the patient’s pronunciation can be documented because the phonetic malformation of the sounds/s/(voiceless “s”),/z/(voiced “s”),/th/and/sh/(“sh” behind e and i) is often a side effect of tongue dysfunction and, thus, MD. Sigmatism (i.e., the phonetic malformation of the sounds/s/and/z/) is the most common pronunciation issue [9]. This occurs as a lisp, with the tongue abutting the anterior teeth (sigmatism addentalis), between the anterior teeth (sigmatism interdentalis), or between the posterior teeth (sigmatism lateralis). These last of these can be bilateral (“bi” is recorded for bilateral), right, or left. If the sound/sch/is malformed, this is called schetism, and the malformation of the/ch/sound is called chitism. Finally, the examination sheet includes the so-called Payne technique to verify and visualize the tongue position during swallowing (Figure 2). For this purpose, the sheet contains an illustration of a mouth with a tongue and palate, as well as the following designated points: R (right edge of tongue), L (left edge of tongue), 1 (tip of tongue), and 2 (center of tongue). The impressions on the palate and/or teeth can be drawn in here. In addition, whether there was contact with the teeth and whether the points were smudged can be recorded. Predominantly blurred points and contact with the teeth indicate orofacial dysfunction. Thus, screening via the Payne technique would be indicated.

Points are awarded for an MD diagnosis on the screening sheet. At the end of the screening sheet, all recorded point values are added together and entered in the appropriate boxes. This can be done in the presence of the patient or without them, depending on whether one wishes to evaluate the result directly with the patient. If a score of 15 is reached, speech therapy is recommended (Figure 2). Figure 1 and Figure 2 shows the interdisciplinary orthodontic-logopedic screening sheet.

### 2.3. Testing the Screening Sheet with Patients

The present study included 108 patients undergoing orthodontic treatment between December 2021 and July 2022. It was irrelevant for the study whether the patients were before, during, or after orthodontic treatment. Patients who had not started or had already completed logopedic treatment were also examined. Patients were referred for an orthodontic control appointment or initial examination and were randomly selected for the study. The patients were examined twice with the orthodontic-logopedic screening questionnaire in the Polyclinic for Orthodontics in the Center for Dental, Oral, and Maxillofacial Medicine of the University Hospital Jena, as well as in two private orthodontic practices. There was a 6-month interval between the initial examination and the follow-up. Patients were properly informed before the beginning of the study about the examinations to be performed, the collection of data, and any need for logopedic therapy or reappearance. The ethics committee of the medical faculty of Friedrich-Schiller-University Jena approved the performance of our investigations (Reg. No.: 2021-2439_BO).

**Figure 3:**
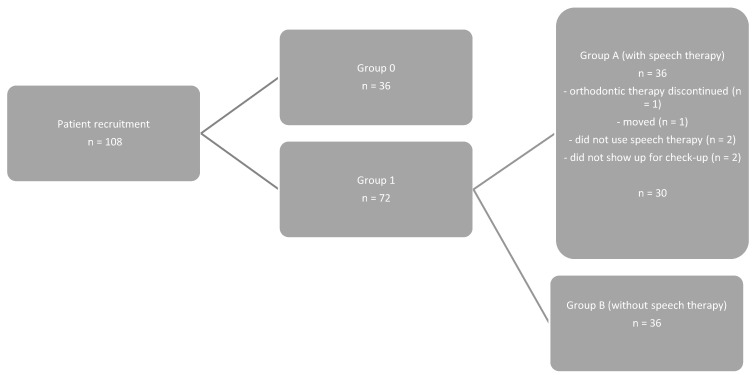
Distribution of the patient collective into the groups.

The 108 patients were examined with the newly devised orthodontic-logopedic screening questionnaire and divided into groups. Group 0 (*n* = 36) had a point score of less than 15 points, and its members were not considered further in the study, as they did not have MD. Group 1 (*n* = 72) included all patients with scores above 15 points who had MD. Group 1 was randomly divided into Subgroup A (*n* = 30), which included those for whom speech therapy was prescribed, and Subgroup B (*n* = 36), which included those for whom speech therapy was not prescribed. The Speech therapy in Subgroup A was prescribed 10 sessions for 45 min per session. Six patients in group A dropped out during the study period for various reasons and were, therefore, not considered further. Group B is the reference group (Figure 3).

### 2.4. Data Analysis and Statistics

For the analysis, anonymized patient data were first tabulated using Excel (Microsoft Office Excel 2021) and consecutively assigned to identification numbers from 1 to 108. The data were then transferred to the statistical program SPSS (IBM SPSS Statistics Version 28.0) and subsequently subjected to detailed descriptive data analysis in collaboration with the Institute of Medical Statistics, Informatics, and Data Science at the University of Jena. For this purpose, the results of the screening forms for the entire patient collective were analyzed after the first follow-up and, among other things, placed in relation to patient age and orthodontic classification.

The effectiveness of the logopedic treatment was assessed using the score on the screening questionnaire after six months. Because no prior information was available regarding the score, the number of cases was estimated using the Cohen’s d effect measure. The questionnaire score at baseline and the difference at six months versus baseline were compared between the two subgroups to be treated (Subgroups A and B) using a two-sided nonparametric Mann–Whitney U test. The median and interquartile range were examined for both groups. Furthermore, the applicability of the screening was analyzed in terms of implementation times between the speech-language pathologist and the non-language pathologists. For this purpose, the nonparametric Wilcoxon signed-rank test for dependent samples was applied, and the median and interquartile range were considered. To examine the influence of age on treatment outcomes, Spearman’s correlation coefficient, with a 95% confidence interval, was calculated. Gender dependence was examined using a two-sided nonparametric Mann–Whitney U test. The significance level was set at *p* = 0.05.

## 3. Results

An interdisciplinary orthodontic-logopedic screening-procedure was developed to evaluate myofunctional dysfunction objectively and in a patient-individualized manner (Figure 1). This questionnaire was tested on 102 patients, and first assessments and results for individualized interdisciplinary orthodontic-logopedic treatment were obtained.

### 3.1. Results of the Patient Examination with the New Orthodontic-Logopedic Screening Procedure

**Figure 4:**
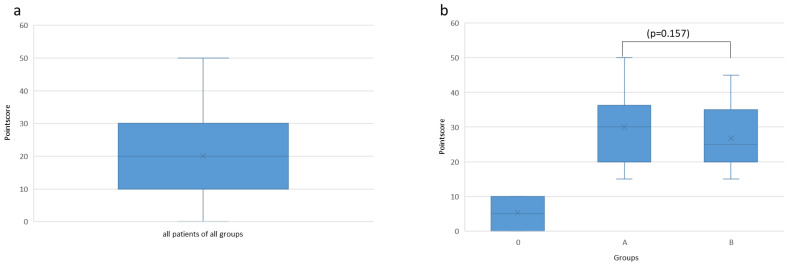
(**a**) Range and median of the point scores of all patients in all groups and (**b**) the point score distribution in the group A (with speech therapy) and group B (without speech therapy) before the start of treatment.

A total of 102 patients between the ages of 6 and 50 years were studied, and 57 male and 45 female patients were included. With a mean age of 13.75 ± 6.91 years and 40 removable and 56 fixed appliances, the cohort represents a typical collective of young patients undergoing orthodontic treatment. First, in accordance with the focus of our study, a score from 0 to a maximum of 50 points could be determined in *n* = 102 patients in the newly developed orthodontic-logopedic screen. The median score was 20, with an interquartile range of 20 (Figure 4). Based on this, patients were divided into Groups 0 and 1. The patients in Group 0 had a median score of 5, with an interquartile range of 10 (Figure 4). The patients in Group 1 (*n* = 66) achieved a score from 15 to a maximum of 50 points in the screening. They were then randomly divided into Subgroups A and B. The median score of Subgroup A was 30.0 (*n* = 30), and that of Subgroup B was 26.81 (*n* = 36). At the median, this manifested itself in a score of 30 for Subgroup A and 25 for Subgroup B, as well as an interquartile range of 16 for Subgroup A and 15 for Subgroup B, respectively. No significant difference (*p* = 0.157) could be found between the two groups at baseline according to the Mann–Whitney U test (Figure 4).

**Figure 5:**
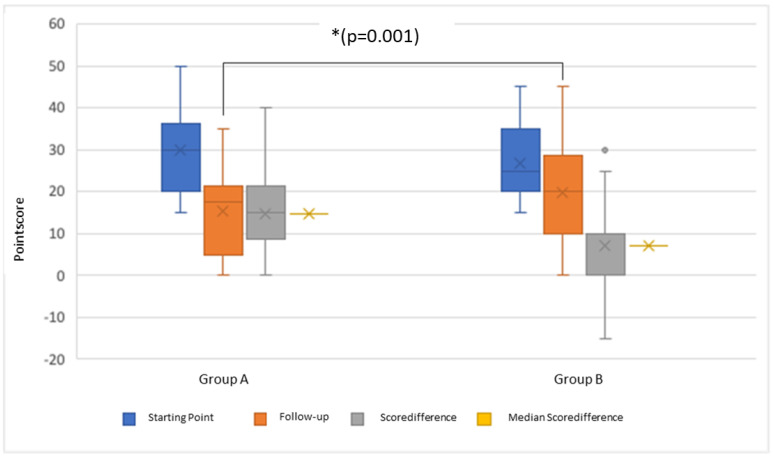
Change in the distribution of the point score between Subgroups A (with speech therapy) and B (without speech therapy) with a significance of * *p* = 0.001.

Subgroup A received speech therapy, in addition to orthodontic treatment, during the treatment interval. Subgroup B received purely orthodontic treatment. To compare the two groups and thus analyze the success of the different treatment approaches, the scores had to first be related to one another. The difference between the score at the starting point (Result 1) and the score at the first follow-up (Result 2) was calculated (Result 1—Result 2). Thus, a large difference represents a large improvement in symptoms at first follow-up. A small difference indicates a small improvement, and a negative difference indicates a worsening of symptoms. Group A patients improved significantly more at the first follow-up as compared to Group B (*p* = 0.001). Group B had a mean difference of 7.08. Group A, on the other hand, had a mean difference of 14.67 in the analysis (Figure 5).

Of the 66 subjects in Subgroups A and B, 40 still showed a need for speech therapy at the first follow-up. Of these, 17/66 belonged to Subgroup A, and 23/66 belonged to Subgroup B. In the exploratory data analysis, Subgroup A achieved a median score of 20, with an interquartile range of 10, and Subgroup B achieved a median score of 25, with an interquartile range of 15 (Figure 5). Furthermore, 26 of 66 patients achieved a score < 15 in the second examination, with these being equally distributed between Subgroups A and B. The 13 subjects in Subgroup A achieved a median score of 0, with an interquartile range of 5. The 13 subjects in Subgroup B, on the other hand, achieved a median score of 10, with an interquartile range of 0. Among the 17 patients in Subgroup A who showed a renewed need for speech therapy, 10 patients were still receiving speech therapy at the first follow-up. This had not yet been completed at the time of the second examination. Seven subjects showed symptoms again, although speech therapy had already ended. Again, all patients in Subgroup A had already completed the planed amount of speech therapy.

Finally, we investigated the extent to which myofunctional dysfunction is correlated with malocclusion and orthodontic findings. For this purpose, the most frequently represented orthodontic indication groups (KIG), according to which the indication for orthodontic treatment in Germany is classified, were analyzed in the study. These included KIG grade M—sagittal step mesial (26); KIG grade D—sagittal step distal (23); KIG grade P—lack of space (9); KIG grade K—transversal deviation 2 (8); and KIG grade U—an undercount of teeth (8). Table 2 shows the KIG of all examined patients and their distribution into individual subgroups. In patients with KIG grades U (37.5%), D (39.1%), and M (34.6%), a similar percentage showed no or only minor symptoms of orofacial dysfunction (score < 15) at baseline. In contrast, significantly fewer patients could be assigned to KIG grades K (12.5%) and P (11.1%) (Table 2).

**Figure 6:**
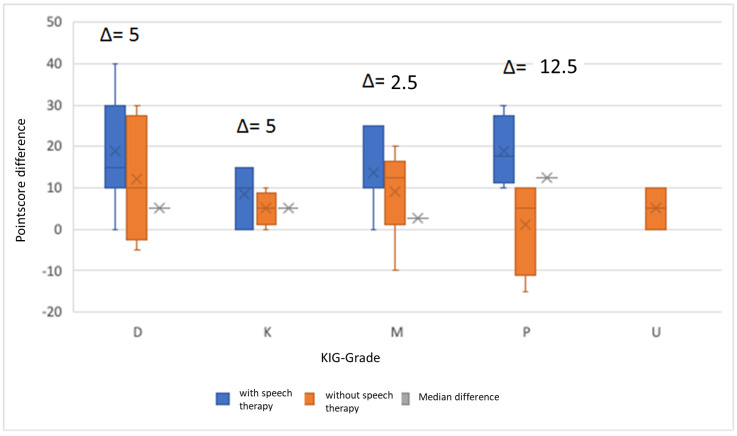
Effectiveness of orthodontic treatment with and without speech therapy by score difference for the five most common KIG grades.

In the exploratory data analysis for the mentioned KIG grades, it was noticed that patients with KIG grades M and K showed the strongest abnormalities, with a median score of 25 on the first examination based on the screening procedure. The interquartile range for patients with KIG grade M was 25, and that for patients with KIG grade K was 18. Patients with KIG grade U showed the mildest symptoms. They achieved a score of 17.5 at the median and an interquartile range of 18. However, no significant difference (*p* = 0.447) can be found between the mentioned KIG at baseline. An overview of the scores at baseline and over the course of treatment for the five most frequent KIGs is shown in Figure 6.

When analyzing the treatment success (the difference between the starting score and the score at the first follow-up) based on KIG classification, there are also no significant differences between the individual KIGs (*p* = 0.139). In the exploratory data analysis, regardless of the treatment method (with speech therapy or orthodontics alone), patients with a distal bite achieved the highest median score difference, 15, with an interquartile range of 26.25, indicating that these patients benefited the most from treatment. The lowest improvement in symptoms was shown by patients with KIG grades of K and U. They each achieved a score difference of 5, with an interquartile range of 10 (Figure 6). Considering only the patients who received speech therapy, patients with a space deficiency in the posterior region (KIG grade P) showed the greatest improvement in symptoms. These achieved a median score difference of 17.5, with an interquartile range of 16.25. The least improvement was seen in patients with KIG grades M and K. These patients achieved a median score difference of 17.5, with an interquartile range of 16.25. They each achieved a median score difference of 10, with an interquartile range of 15 (Figure 6). Among patients who received purely orthodontic treatment, patients with a mesial bite showed the greatest treatment success (Figure 6).

### 3.2. Applicability of the Orthodontic-Logopedic Screening Procedure

**Figure 7:**
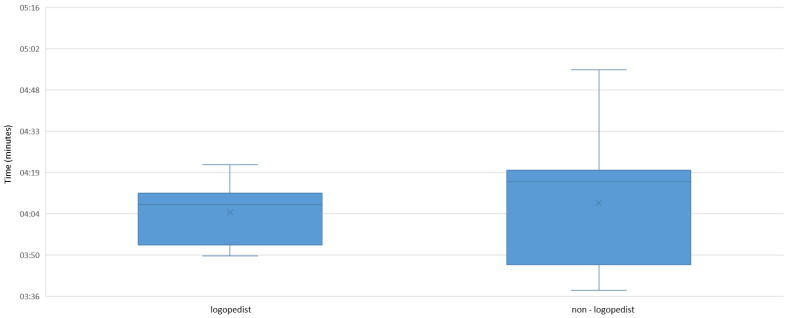
Applicability of the orthodontic-logopedic screening procedure in terms of time.

In addition, the applicability of the newly developed orthodontic-logopedic screening questionnaire was examined. For this purpose, the time taken by orthodontists (hereafter referred to as non-logopedists) to perform the examination was compared with that of a doctoral student of dentistry with logopedic training and professional experience (hereafter referred to as the logopedist). Only situations in which two examiners examined the same patient were compared. Exploratory data analysis showed that a non-logopedist took a median of 4:16 min (interquartile range 0:33 min) to perform the entire screening on the patient. Only slightly less time was taken by the speech therapist with a median of 4:08 min (interquartile range 0:18 min) (Figure 7). No statistically significant Wilcoxon rank test results (*p* = 0.735) could be found. Thus, there was no significant difference the screenings performed by a speech therapist versus a non-logopedist.

## 4. Discussion

The causal relationship between dysgnathia and myofunctional dysfunction is generally known and already well researched [2,10,11,12]. In order to optimally and successfully treat patients with malocclusion and MD, cooperation between the departments of orthodontics and speech therapy is essential [13,14]. Nevertheless, inadequately treated or untreated orofacial dysfunctions, as well as those detected late or not at all, still lead to prolonged orthodontic treatment duration and/or recurrences [10,11]. Thus, Saccomanno et al. (2012) describe the correct diagnosis of MD and the right timing for interdisciplinary, patient-specific, and personalized therapy as crucial to treatment success [13]. In order to simplify the diagnosis and find the right time for supportive speech therapy in the orthodontic daily routine [10,15], an interdisciplinary screening procedure that can objectify the need for speech therapy would be helpful for diagnosers [14,16]. However, something like this is difficult to find in the literature. As Korbmacher et al. (2004) also note, there is currently a lack of standardized and specialized diagnostics for myofunctional dysfunction. Moreover, due to the lack of reproducible documentation and progression diagnostics, scientific evidence on the effectiveness of MD treatment is hampered [4]. For this reason, scientific research and the expansion of interdisciplinary diagnostics, communication, and collaboration for the complex of orofacial dysfunctions are of particular importance.

In the pilot study presented here, with a mean age of 13.75 years and a standard deviation of 6.91 years, the cohort represents a typical collective of young patients undergoing orthodontic treatment. In a similarly designed study from 1997 that investigated the effectiveness of MD treatment for an open bite or enlarged overjet in terms of improving dental occlusion, the patient cohort had a similar mean age of 14.19 ± 7.87 years [10]. In addition, several studies have shown that patients with any form of malocclusion can have orofacial imbalances [10,12,15,17]. The cross-sectional study conducted by Paolantonio et al. (2019) examined the prevalence of oral habits and mouth breathing as important representatives of orofacial dysfunction, as well as the presence of various malocclusions in children aged 3 to 6 years. More than half (54%) of the children who required orthodontic treatment also had one of these two risk factors. A significant association was found between orofacial dysfunctions and malocclusions, such as an open bite, a crossbite, an enlarged overjet, and severe dental malocclusions, as was the case with Grippaudo et al. (2016) in children older than 6 years [12,18]. For this reason, patients with various degrees of KIG and with both removable and fixed appliances were studied. There were no exclusion criteria in this regard. Thus, it can be assumed that the collective we examined corresponds to a real patient collective that is undergoing orthodontic treatment and may exhibit orofacial dysfunctions. The total collective was divided into the different subgroups using the score developed specifically for the screening sheet. Only Grandi’s (2012) examination protocol has a similar distribution of scores for the quick, easy detection of orofacial dysfunction and morphological changes [16]. However, in our view, this does not provide the desired focus on the diagnosis of orofacial dysfunction and should, rather, be seen as an initial indicator of orofacial dysfunction. In addition, the point system primarily serves to assign patients to appropriate specialists, not to objectify the dysfunction pattern and, thus, a potential follow-up [16]. By randomly dividing Group 1 (score ≥ 15; 66/102 patients) into Subgroup A (with external speech therapy; 30/102 patients) and Subgroup B (without speech therapy; 36/102 patients) as a control group, we addressed an important aspect of the scientific study of orofacial dysfunctions, as well as their treatment in cases of malocclusion. Because our study design only provides for a delay in speech therapy treatment of approximately six months, rather than a morally and ethically indefensible omission of therapy, the major research dilemma involved in investigating the effectiveness of MD treatment in diagnosed dysfunctions using a control group can be circumvented in this way [10]. A following speech therapy after the study for all individuals who were in need of it was, of course, available and provided. This represents another important step called for in previous studies [10,19] in the spirit of evidence-based and personalized medicine.

The aim of the study was to diagnose patients with MD more easily and to treat them more effectively using the newly developed orthodontic-logopedic screening procedure. We investigated two treatment approaches. Some of the patients diagnosed with orofacial dysfunction received combined orthodontic-logopedic treatment (Group A), and others received purely orthodontic treatment (Group B). The patients in Subgroup A (mean score difference of 41.62 points) improved significantly at the first follow-up as compared to the patients in Subgroup B (mean difference of 26.74 points; *p* = 0.001). This shows that patients benefit from combined orthodontic-logopedic therapy and confirms the results of Van Dyck et al. (2016) and Benkert (1997) [10,19]. In Van Dyck et al. (2016), the improvement of the group with logopedics, as compared to the group without logopedics, was expressed in particular by a significant improvement in maximum tongue pressure, tongue rest position, the swallowing of solid food, and the contact of the lower incisors with their antagonists or the palate at follow-up (Van Dyck et al. 2016) [19]. Research by Benkert (1997) also found that orthodontic treatment with additional speech therapy had a positive effect on dental and maxillary malocclusions [10]. Thus, logopedics can be considered a useful adjunct to orthodontics in the presence of orofacial dysfunction. Moreover, the screening sheet turns out to be a useful means of diagnosing and documenting this. The limitations of the study are that the patients were randomly selected and, therefore, there could not be an equal distribution of malocclusions and orofacial dysfunctions. These have then been more difficult to compare among themselves. Because all malocclusions were considered in our case, it was logical to focus the scoring on orofacial symptoms. However, it must be discussed as a limiting factor of the present study that the patients in Subgroup A may have shown improved symptomatology only in a clinical situation. Because the study was not blinded, the patients in the second examination knew what was required of them and were able to recall the patterns learned in therapy. Thus, the transfer of what was learned to everyday life is not guaranteed. Van Dyck et al. (2016) also come to a similar conclusion [19]. Nevertheless, it can be assumed that the patients pay more attention to the correct lip/tongue position—depending on the weak point—as a result of their acquired knowledge, and thus, the improved symptoms can be integrated more easily in everyday life. Furthermore, we were able to show that the patients in Subgroup B also benefited from their therapy approach, although this occurred to a lesser extent than with Subgroup A. This was because, after the six-month therapy phase, the same number of patients in Subgroups A (*n* = 13) and B (*n* = 13) no longer required speech therapy. This shows that the improvement of the malocclusion alone can lead to an improvement of the symptoms and confirms the close connection between malocclusion and MD. Nevertheless, patients with MD who do not receive speech therapy should be monitored closely. The comparison of the orthodontic findings/orthodontic indication groups (KIGs) with the occurrence and presence of MD confirms that individual KIG grades are more frequently associated with MD and that these are correlated with one another. Likewise, interdisciplinary, personalized orthodontic-logopedic combination therapy is more effective for the therapeutic success of individual KIG grades. In order to be able to make a meaningful statement on this topic, the case numbers presented for the individual KIG grades are not sufficient. Further studies to confirm the hypothesis are necessary.

Regarding the applicability of our screening, we did not find a statistically significant difference in implementation time between the non-logopedists and the speech therapist (*p* = 0.735). Thus, the application of the screening can be considered independent of logopedic expertise. Moreover, only the times for the same subject when the screening was performed by two different examiners were included in the analysis. For this reason, the results prove to be independent of the patient’s age and compliance. With median times of 4:16 min (non-logopedist) and 4:08 min (logopedist), the target execution time of five to seven minutes was met by both disciplines. In their “Proposal for an interdisciplinary diagnostic questionnaire for orofacial dysfunctions”, Korbmacher et al. (2004) emphasized the importance of fast, reproducible documentation. On average, the speech therapists needed five minutes or less to record the diagnostic questionnaire, which was compressed onto one A4 page. They describe this time as “also feasible in everyday practice” [4]. No time-related guidelines are available for the comparable screening questionnaires provided by Grandi (2012) and de Felicio et al. (2008) [16,20]. They are only described as simple and quick clinical procedures. Only for the “Expanded protocol of orofacial myofunctional evaluation with scores” (OMES-E), created by de Felicio et al. (2010), do the authors indicate a total execution time of 15 min [21]. We also see an improvement in the precise recording and documentation of both the orthodontic parameters and the symptoms of MD, as well as in the assignment of points for the essential symptoms. Thus, the practitioners not only receive an overview of all symptoms but also an objectified and comparable decision-making aid regarding speech therapy, as well as a progress documentation. This serves not only the optimization of patient care and thus improve the quality of life but also as documentation in the sense of a personalized evidence-based medicine [22]. This is where the presented orthodontic-logopedic screening procedure stands out from all comparable screening forms and shows clear improvement.

## 5. Conclusions

This is one of the first studies to develop an interdisciplinary, orthodontic-logopedic screening procedure for myofunctional dysfunction and test its feasibility. Thus, it represents an important step in diagnostics and therapy for individual orthodontic treatment. It is based for the first time on various occlusion disorders as well as a wide range of myofunctional dysfunctions. Furthermore, it proves the applicability for orthodontists as well as speech therapists in a well practicable time interval. The screening sheet is available to interested orthodontists and speech therapists.

## Figures and Tables

**Table 1:** Literature and sources for the development of the screening sheet [1,4,5,6,7,8].

	Result Number	Name/Author of Used Examination Forms
Database ThULB	1	- Speech therapy brief findings for myofunctional disorders/I. Adamer, M. Furtenbach, Dr. A. Schwarz
PUBMED	1	- Proposal for an interdisciplinary diagnostic questionnaire for orofacial dysfunctions/H.Korbmacher, G. Böhme, A. Kittel, B. Kahl-Nieke
madoo.net	6	- MD—survey/A. Makdissi - Myfunctional disorders/S. Krechting
Specialist books, therapy material for logopedic practice	6	- Diagnostic sheet swallowing process/S. Bauer - Anamnesis/diagnostics MD/A. Kittel - Cologne diagnostic sheet for myofunctional disorders/B. Giel, M. Tillmanns-Karus - Tübingen survey form for clinical-functional findings prior to orthodontic treatment/N. Schwenzer

**Table 2:** Distribution of the orthodontic findings/orthodontic indication group (KIG) for the entire patient collective.

		Subgroups			
Anomaly (Grade)		0	A	B	Total
None according to statutory health insurance	Number in %	3 50.0%	1 16.7%	2 33.3%	6 100.0%
Aesthetic treatment (private)	Number in %	2 40.0%	0 0%	3 60.0%	5 100.0%
Craniofacial anomaly (A)	Number in %	0 0.0%	0 0.0%	2 100.0%	2 100.0%
Tooth underscore (U)	Number in %	3 37.5%	0 0.0%	5 62.5%	8 100.0%
Breakout disorder (S)	Number in %	2 40.0%	1 20.0%	2 40.0%	5 100.0%
Sagittal step distal (D)	Number in %	9 39.1%	9 39.1%	5 21.7%	23 100.0%
Sagittal step mesial (M)	Number in %	9 34.6%	11 42.3%	6 23.1%	26 100.0%
Vertical step open (O)	Number in %	0 0.0%	1 50.0%	1 50.0%	2 100.0%
Vertical step deep (T)	Number in %	1 100.0%	0 0.0%	0 0.0%	1 100.0%
Transversal deviation 1—bilateral crossbite (B)	Number in %	2 66.7%	0 0.0%	1 33.3%	3 100.0%
Transversal deviation 2—unilateral crossbite (K)	Number in %	1 12.5%	3 37.5%	4 50.0%	8 100.0%
Narrow stand front (E)	Number in %	3 75.0%	0 0.0%	1 25.0%	4 100.0%
Shortage of space page (P)	Number in %	1 11.1%	4 44.4%	4 44.4%	9 100.0%
Total	Number in %	36 35.3%	30 29.4%	36 35.3%	102 100.0%

## Data Availability

Raw data are available from the corresponding author upon reasonable request.
